# Genome-Wide Identification of the *KAN* Gene Family and Expression Profiles During the Fruit Developmental Stages in *Prunus mume*

**DOI:** 10.3390/ijms26189121

**Published:** 2025-09-18

**Authors:** Minglu Li, Xiao Huang, Ximeng Lin, Ziqi Wang, Feng Gao, Zhihong Gao

**Affiliations:** Laboratory of Fruit Tree Biotechnology, College of Horticulture, Nanjing Agricultural University, Nanjing 210095, China

**Keywords:** *Prunus mume*, *PmKAN*, gene expression, bioinformatics

## Abstract

*KANADI* (*KAN*) transcription factors are pivotal regulators of lateral organ polarity establishment in plants. Although extensively studied in herbaceous plants, the role of *KAN* genes in woody plant development remains unclear. This study conducts the first comprehensive analysis of 26 *PmKAN* genes in *Prunus mume*, elucidating their evolutionary trajectories, structural configurations, tissue-specific expression patterns and potential roles in root and fruit development. Phylogenetic analysis of four Rosaceae species and *Arabidopsis thaliana* clustered these *PmKANs* into five subfamilies, with conserved motif patterns supporting this classification. Chromosomal localization revealed that all *PmKAN* members are distributed across eight chromosomes, with tandem duplications events and syntenic relationships indicating functional diversification driven by gene family expansion. Cis-regulatory element analysis identified light-responsive, hormone-associated, stress-related, and developmental motifs, suggesting *PmKAN* genes are involved in regulating plant physiological processes and development. The qRT-PCR analysis revealed tissue-specific expression heterogeneity among *PmKAN* genes, with markedly elevated expression particularly observed in roots and fruits. Further expression profiling across fruit developmental stages suggests potential stage-specific functional divergence of *PmKAN* genes during fruit development. This study provides a theoretical foundation for further investigating the evolutionary relationships and molecular regulatory mechanisms of the *PmKAN* gene family.

## 1. Introduction

The *KANADI* gene family, belonging to the GARP subfamily of transcription factors under the MYB superfamily, exhibits highly conserved characteristic domains [1]. Initially identified in the model plant *Arabidopsis thaliana*, this family comprises four members (*AtKAN1-4*) characterized by a genomic structure containing six exons and a single open reading frame (ORF) of 403 amino acids (predicted molecular weight: 45.8 kDa), featuring a 56-amino acid GARP conserved domain with two histidine residues [1,2]. Subsequent investigations have revealed *KAN* orthologs in diverse plant species including *Zea mays* [3], *Oryza sativa* [4], *Nicotiana benthamiana* [5], and *Populus trichocarpa* [6]. The evolutionarily conserved GARP domain facilitates critical biological processes such as chloroplast differentiation, cytokinin signalling transduction, phosphorus metabolism, and organ polarity establishment [7]. At the same time, recent studies have documented the existence of this gene family in spore-bearing plants like ferns [8,9], current research predominantly focuses on herbaceous model species. However, functional characterization in woody plants remains largely unexplored, representing a significant knowledge gap in understanding the full spectrum of *KANADI* gene functions across plant taxa.

Previous studies indicate that the *KANADI* (*KAN*) genes are specifically expressed in the distal differentiation zone of lateral organs and in the phloem tissues of the vascular system during early plant development [10,11]. These genes regulate polarity establishment by maintaining abaxial identity while suppressing adaxial characteristics [2]. The *Arabidopsis thaliana* genome harbours four functionally redundant *KAN* paralogs (*KAN1-4*), where single-gene mutants display negligible phenotypic deviations, whereas double or triple mutants exhibit severe abaxial polarity defects. For instance, *KAN1*/*KAN2* double mutants develop narrow leaves with abaxial protrusions and adaxialised floral organs, while *KAN1*/*KAN2*/*KAN3* triple mutants produce mature leaves with multi-planar expansion, forming elongated blades with fan-shaped distal regions [1,12]. These observations confirm substantial functional redundancy within the *Arabidopsis thaliana KAN* family. In the monocots, the rice *KAN* ortholog S*HALLOT-LIKE1* (*SLL1*) localizes specifically to leaf abaxial domains. *SLL1* loss-of-function mutants exhibit abaxial polarity loss, triggering mesophyll cell apoptosis alongside ectopic adaxial features such as chloroplast overaccumulation and enhanced photosynthesis. In contrast, *SLL1* overexpression enhances phloem development, suppresses palisade tissue formation, and induces dwarfism with leaf curling [4]. Similarly, maize *MILKWEED POD1* (*MWP1*) regulates dorsoventral polarity, as evidenced by the pronounced abaxialisation in dominant *mwp-R* gain-of-function mutants [13].

The *KANADI* gene family is well-established as a critical regulator of polarity establishment in vegetative organs such as leaves and roots, while its roles in other developmental traits remain under investigation. Molecular studies demonstrate that *KANADI* transcription factors directly bind target loci to regulate abaxial identity. For instance, *OsKANADI1* in rice (*Oryza sativa*) interacts with promoters of grain size-related genes, suggesting regulatory roles in seed morphogenesis [14]. Mechanistic investigations reveal *OsKANADI1* binds the intronic region of *OsARF3a*, synergizing with tasiR-ARFs pathways to maintain *OsARF3a* expression levels for proper lemma development [15]. Recent findings show *Oskan1* mutants exhibit constitutive semi-dwarfism with shortened internodes. Molecular profiling identifies *OsKAN1* as a transcriptional repressor of *OsYAB5*, forming a functional *OsKAN1*-*YAB5* complex that suppresses *OsGA2ox6* expression, thereby reducing bioactive gibberellin levels and inhibiting cell elongation [16]. Current research on the *KAN* gene family has primarily focused on herbaceous plants, while studies on its role in the growth and development of woody plants remain limited, with the underlying molecular mechanisms still poorly understood.

*Prunus mume (P. mume*) is a member of the Rosaceae (*Prunus* L.) fruit tree native to southwestern China, and has been cultivated in East Asia (including Japan) for over seven millennia. Its fruits contain substantial amounts of minerals and bioactive compounds, demonstrating significant pharmaceutical and commercial potential [17]. Recent advancements in molecular biology and bioinformatics have enabled genome-wide identification and functional characterization of plant genes [18,19]. Although extensive studies on *KAN* genes have been conducted in various species, a systematic analysis of this gene family in *P. mume* remains unexplored. In this study, we employed a combination of bioinformatic approaches to identify and characterize the *KAN* gene family in *P. mume*. Furthermore, qRT-PCR was utilized to analyze the tissue-specific expression patterns of *PmKAN* genes. This work aims to establish a foundation for further investigation into the biological functions of these genes.

## 2. Results

### 2.1. Identification of KAN Family Members in P. mume Genome

**Figure 1:**
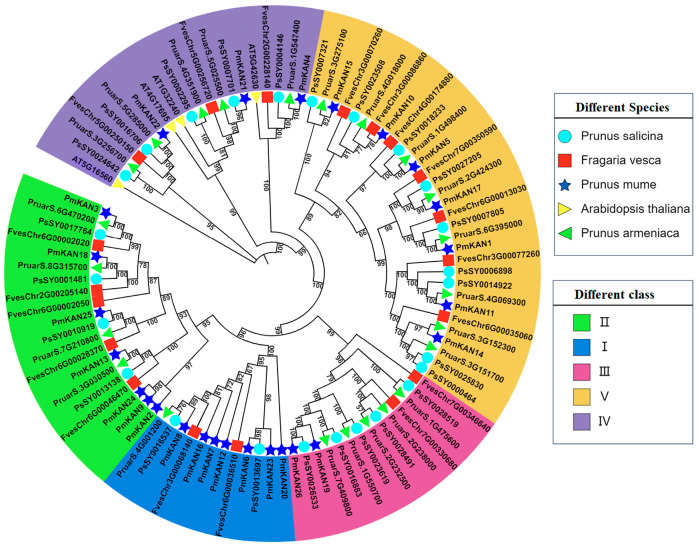
Phylogenetic analysis of KAN proteins from *Prunus mume* and four other plant species.

Using IQ-TREE, a phylogenetic tree was constructed from the amino acid sequences of 97 *KAN* genes. This dataset comprised 26, 4, 19, 25, and 23 *KAN* genes from *P. mume*, *A. thaliana*, *F. vesca*, *P. salicina*, and *P. armeniaca*, respectively. Based on sequence similarity, these proteins, including the 26 *PmKANs* from *P. mume*, were categorized into five groups (Figure 1). The distribution of *PmKAN* genes varied across these subgroups: Group I, II, and V contained the highest number (7 representatives each), while Group III had the fewest (2 representatives). All *PmKAN* family members clustered closely with homologous genes from the other three Rosaceae species, indicating a close evolutionary relationship and suggesting potential functional conservation.

Through systematic filtering using PlantTFDB to eliminate duplicate sequences and those lacking DNA-binding domains (DBD), 26 *KAN* family members (*PmKAN1*–*PmKAN26*) were identified in *P. mume*, as catalogued in Table 1. Comprehensive physicochemical profiling revealed significant molecular divergence: amino acid lengths spanned 240 residues (*PmKAN10*/*15*) to 496 residues (*PmKAN18*/*26*), with molecular weights ranging from 26.7 kDa (*PmKAN10*) to 54.3 kDa (*PmKAN26*). The isoelectric points (pI) ranged from 5.09 for *PmKAN16* to 9.16 for *PmKAN2*. About 61.54% (16/26) had a pI above 7.0, showing that most PmKAN proteins are basic in nature. Instability index values went from 37.42 for *PmKAN20* to 75.71 for *PmKAN13*. Aliphatic index values varied from 56.94 for *PmKAN26* to 81.03 for *PmKAN7*.

**Figure 2:**
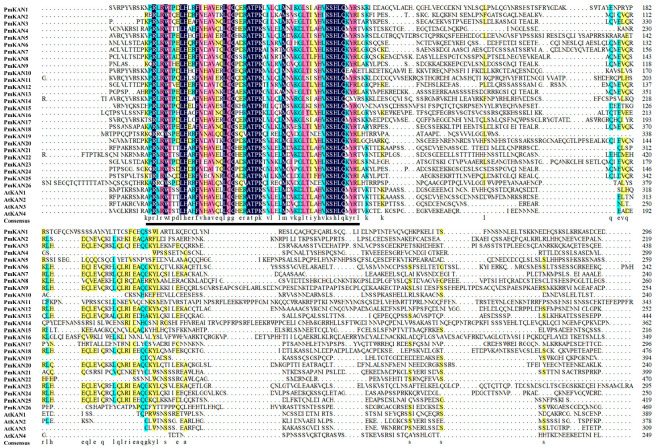
Amino acid sequence comparison of KAN in *P. mume* and *A. thaliana*. Colors indicate the proportion of homologous sequence similarity (black: 100%; pink: ≥75%; green: ≥50%; yellow: ≥33%); sequences with similarity below 33% are shown without background color. The underlined region indicates the conserved GARP domain.

Hydrophobicity analysis (GRAVY scores: −1.06 to −0.352) confirmed that 96.2% (25/26) of *PmKANs* were hydrophilic (<−0.5), with only *PmKAN7* (−0.352) exhibiting amphipathic properties. Collectively, these findings demonstrate that most *P. mume* KAN proteins are alkaline, hydrophilic, and structurally unstable. Sequence alignment of 30 KAN proteins (26 from *P. mume* and 4 from *A. thaliana*) revealed the conserved presence of a canonical GARP domain (Figure 2).

### 2.2. Secondary Structure Analysis of PmKAN Protein

**Figure 3:**
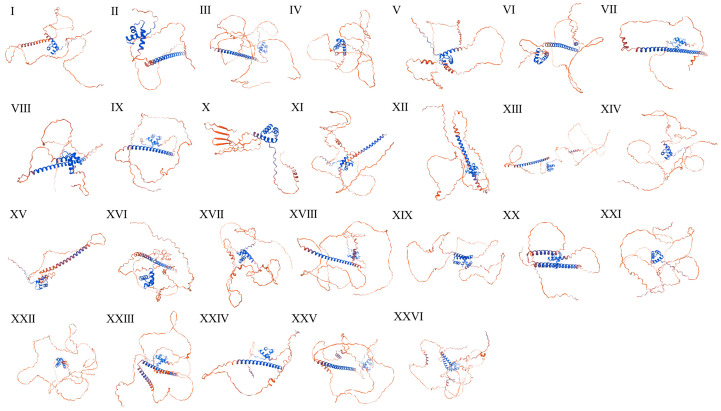
Predicted tertiary structure of *PmKAN* proteins in *Prunus mume*. Blue regions represent α-helices, and red regions represent random coils. Note: I to XXVI represent *KAN1* to *KAN26*, respectively.

Secondary structure analysis of *P. mume* KANADI proteins (Table 2) identified five structural elements: coil regions, 310 helices, α helix, random coils, β Strand and β turn. Coil regions (68.49~90.85%) and α helices (6.81~28.62%) dominated the structural composition, while other elements each accounted for <5%. The prediction of tertiary structures for 26 KANADI proteins using SWISS-MODEL revealed conserved structural architectures dominated by coil regions and α helices, consistent with secondary structure predictions (Figure 3). Significantly, proteins within the same subfamily exhibited similar secondary structure proportions and partial structural homology, though conformational variations were observed. These structural divergences, potentially arising from differences in element length and spatial arrangement, may underlie functional differentiation.

### 2.3. Gene Structures, Protein Conserved Domain and Motif Compositions of PmKAN Proteins

**Figure 4:**
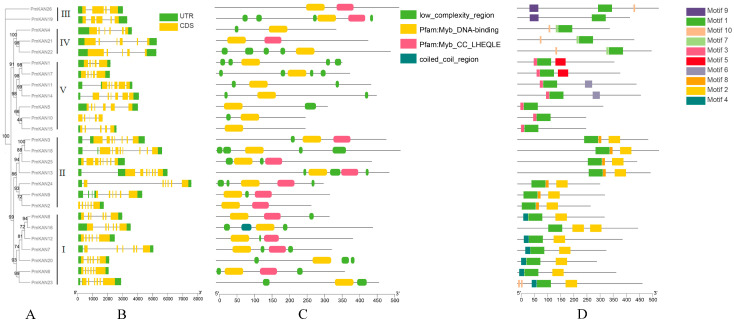
The conserved protein domains, gene structures and conserved motifs of *PmKAN* genes are based on phylogenetic relationships. (**A**) Phylogenetic tree. Maximum likelihood phylogenetic tree constructed using full-length KAN protein sequences from *P. mume* and related species. (**B**) The *PmKAN* structures. The gene elements sizes were comparable to their sequence lengths, and the exons (coding sequences, CDS) depicted as yellow rectangles and untranslated regions (UTRs) shown as green rectangles. (**C**) Protein conserved domains. (**D**) The *PmKAN* conserved motifs. The conserved motifs of the *PmKAN*s and the 10 motifs are displayed in different colours.

To investigate the structural diversity of *KAN* genes in *P. mume*, we performed an analysis of the gene structure (Figure 4). Structural analysis of *PmKAN* genes (Figure 4B) revealed exon numbers ranging from 4 to 8. While *PmKAN26* contained four exons, *PmKAN18* and *PmKAN25* possessed a maximum of eight. Fifteen members (*PmKAN4*, *PmKAN21*, *PmKAN11*, *PmKAN14*, *PmKAN5*, *PmKAN24*, *PmKAN2*, *PmKAN9*, *PmKAN8*, *PmKAN16*, *PmKAN7*, *PmKAN12*, *PmKAN20*, *PmKAN6*, and *PmKAN23*), constituting over half of the family, exhibited six exons. Seven-exon configurations were observed in *PmKAN3* and *PmKAN13*. Most *PmKAN* members maintained both 5′UTR and 3′UTR regions critical for mRNA stability and microRNA interaction, except *PmKAN2*, which lacked the 5′UTR. Remarkably, *PmKAN10* displayed intron-less architectures, suggesting evolutionary conservation.

Domain characterization demonstrated that, as members of the MYB gene family, all *PmKAN* members contain the Myb-DNA-binding conserved domains (Figure 4C). Class I and II members additionally harboured Myb-CC_LHEQLE domains, with coiled-coil regions exclusively present in *PmKAN16*. MEME/TBtools analyses identified ten conserved motifs (motif1–motif10) within 500 amino acid sequences (Figure 4D). Most *PmKAN* genes contained 2–5 motifs, showing no direct correlation between exon structure and subfamily classification. However, phylogenetic analysis showed that certain motif patterns were conserved. Motif1 was found in all proteins. Motif9 was specific to Class III (*PmKAN19* and *PmKAN26*), motif10 was found in Class IV (*PmKAN4*, *PmKAN21*, and *PmKAN22*), and motif3 was typical of Class V (*PmKAN1*, *PmKAN17*, *PmKAN11*, *PmKAN14*, *PmKAN5*, *PmKAN10*, and *PmKAN15*). Classes II and IV shared similar motif patterns, suggesting a close evolutionary link, while Classes I, III, and V had distinct patterns, pointing to different evolutionary paths.

### 2.4. Chromosomal Distributions Analysis of PmKAN Genes

**Figure 5:**
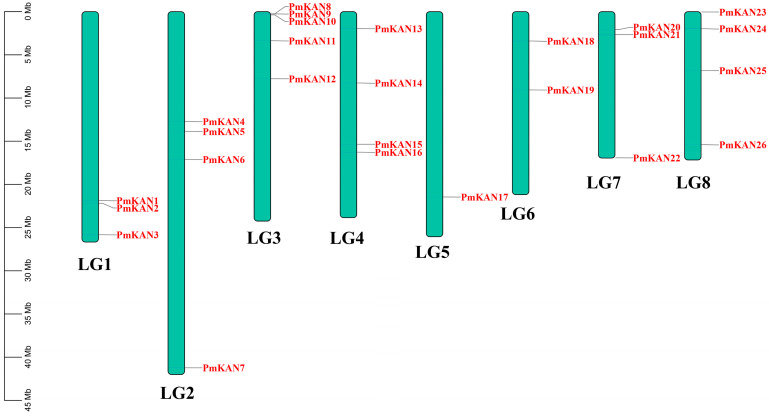
Chromosomal locations of *PmKAN* genes on *P. mume* chromosomes. The scale on the left represents the length of the chromosome. The chromosome numbers are on each chromosome.

Chromosomal localisation significantly influences gene functional dynamics. Our analysis revealed uneven distribution of 26 *PmKAN* genes across eight chromosomes in *P. mume* (Figure 5). Chromosome 3 exhibited the highest gene density with five loci, followed by chromosomes 2/4/8 (four genes each), chromosomes 1 and 7 (three each), chromosome 6 (two), and chromosome 5 containing a single locus. Three duplication types (whole-genome, segmental, tandem) were identified, with tandem duplications defined as chromosomal regions ≤ 200 kb containing ≥ 2 genes. And we find a tandem duplication cluster (*PmKAN8*–*10*) on chromosome 3, which suggests evolutionary expansion through genomic duplication events.

**Figure 6:**
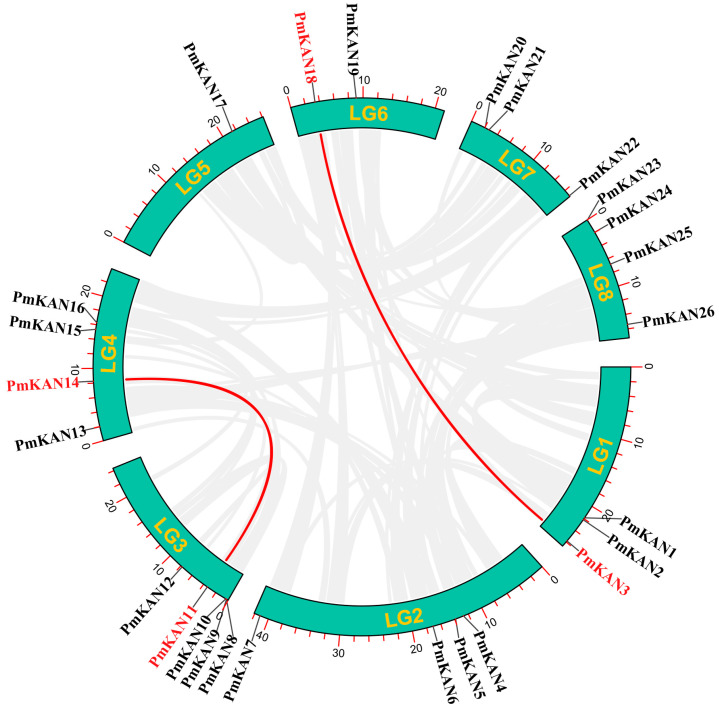
The distribution of the *PmKAN* genes on the chromosomes and syntenic relationships. Chromosomal coordinates (megabase scale) are displayed with yellow numeric identifiers. Paralogous gene pairs exhibiting segmental duplication are connected via red linkage curves.

**Figure 7:**
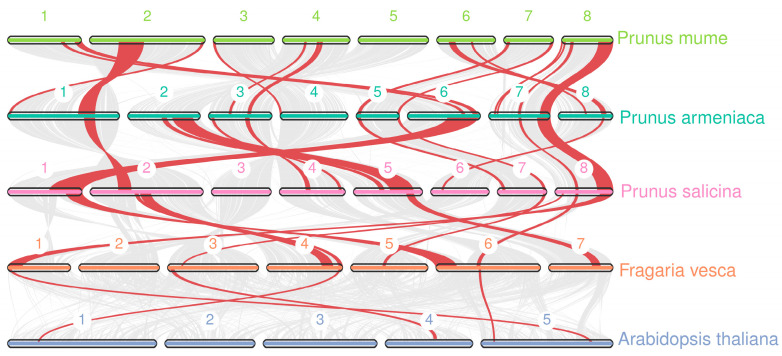
Synteny analysis of *KAN* genes between *P. mume* and other plants. The red line showed the *KAN* syntenic gene pairs. The numbers represent the chromosome numbers of each species.

This study first employed MCScanX software to conduct intragenomic analysis of *PmKAN* genes and interspecies comparisons with *Arabidopsis thaliana KAN* homologs. The intragenomic analysis identified two tandem duplication pairs (*PmKAN3*/*PmKAN18* and *PmKAN11*/*PmKAN14*), belonging to Groups II and V, respectively (Figure 6). Subsequently, to further investigate the duplication mechanisms of *PmKAN* genes, we performed genomic synteny analysis between *P. mume* and its close relatives (*P. armeniaca*, *P. salicina*, *F. vesca*) as well as the outgroup species *A. thaliana* (Figure 7). Extensive syntenic blocks were observed among Rosaceae species compared to *A. thaliana*, particularly within the *Prunus* genus (*P. mume*, *P. salicina*, *P. armeniaca*), indicating conserved genomic architecture and close evolutionary relatedness. The syntenic regions in Rosaceae typically harboured multiple *KAN* paralogs, whereas the corresponding regions in *A. thaliana* usually contained only a few *KAN* orthologs. This pattern suggests that the *KAN* gene family underwent extensive expansion in the common ancestor of Rosaceae. Whole-genome duplication (WGD) events are inferred as the predominant mechanism driving this expansion, with a substantial number of the duplicated *KAN* genes subsequently retained through selective pressure within the Rosaceae lineage.

### 2.5. Cis-Acting Element Analysis in PmKAN Promoter Region

**Figure 8:**
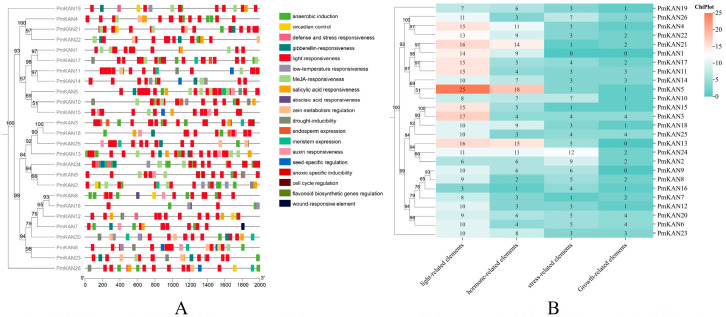
Analysis of cis-acting elements in promoters of *PmKAN* genes. (**A**) The 2000 bp sequences upstream of the 26 *PmKAN* genes were analysed with PlantCARE. (**B**) Types and quantities of cis-acting elements in the promoter region of the *PmKAN* genes.

Transcriptional regulation in plants is critically mediated by cis-regulatory elements within promoter regions. To elucidate the transcriptional mechanisms of *PmKAN* genes, we analysed 2000 bp upstream sequences of 26 *PmKAN* coding regions. Four functional categories of cis-acting elements were identified (Figure 8): light-response-related elements constituted the most abundant group with 303 entities, representing 47.42% of total regulatory components. Phytohormone-associated motifs ranked second, comprising 179 elements including 66 abscisic acid responsiveness, 64 MeJA responsiveness, 19 salicylic acid responsiveness, 17 gibberellin responsiveness, and 13 auxin responsiveness types. Stress-related elements totalled 107 units, spanning anaerobic/drought/low-temperature inducible, defense and stress activated, wound-responsive and anoxic specific inducibility. Development-related components were the least represented category with 50 elements functionally linked to meristem expression, seed-specific regulation, circadian control, and flavonoid biosynthetic genes regulation. Significantly, *PmKAN5* displayed the highest cis-element density among family members with 47 regulatory units, whereas *PmKAN7* and *PmKAN16* uniquely possessed fewer than 15 elements each. These results underscore the multifaceted regulatory potential of *PmKAN* genes in plant growth and metabolism.

### 2.6. Expression Analysis of PmKAN Genes in Different Tissues

**Figure 9:**
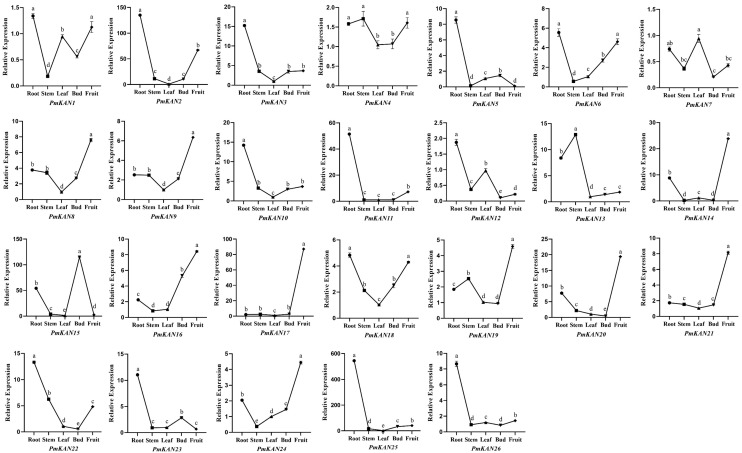
Expression levels of *PmKAN* genes in five different tissues. The x-axis represents different tissue types, while the y-axis indicates the relative expression level of the target gene mRNA. Values are shown as mean ± SE (*n* = 3). Different lowercase letters indicate significant differences (*p* < 0.05) in the expression of *KAN* genes among various tissues of *P. mume*.

To further investigate the tissue-specific expression patterns of the 26 *PmKAN* genes, we examined their expression levels in root, stem, leaf, bud, and fruit tissues using qRT-PCR (Figure 9). The results revealed that the root contained the highest number of genes with elevated expression, totaling 13 genes (*PmKAN1*, *PmKAN2*, *PmKAN3*, *PmKAN5*, *PmKAN6*, *PmKAN10*, *PmKAN11*, *PmKAN12*, *PmKAN18*, *PmKAN22*, *PmKAN23*, *PmKAN25*, and *PmKAN26*). Nine genes showed high expression in fruit (*PmKAN8*, *PmKAN9*, *PmKAN14*, *PmKAN16*, *PmKAN17*, *PmKAN19*, *PmKAN20*, *PmKAN21*, and *PmKAN24*), while two genes were highly expressed in stem (*PmKAN4* and *PmKAN13*). Only one gene each exhibited high expression in leaf (*PmKAN7*) and bud (*PmKAN15*). These findings indicate that *PmKAN* genes display distinct tissue-specific expression patterns, suggesting they may play specialized roles in different tissues of *P. mume*.

### 2.7. Expression Analysis at Different Developmental Stages of Fruit

**Figure 10:**
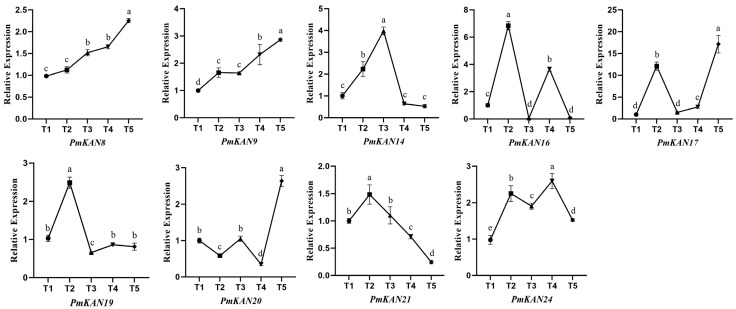
Expression analysis of the *PmKAN* genes in different periods of fruit development. The x-axis represents different periods, while the y-axis indicates the relative expression level of the target gene mRNA. Values are shown as mean ± SE (*n* = 3). Different lowercase letters indicate significant differences (*p* < 0.05) in the expression of *KAN* genes among various development stages of fruit.

Analysis of gene expression levels across different tissues indicated that a considerable number of highly expressed genes within the *PmKAN* family were enriched in root and fruit tissues. Previous studies on *KAN* genes have highlighted their crucial regulatory roles in the polar development of lateral organs such as roots and leaves [2,10,11]; however, their functions in reproductive organs like fruits remain largely unexplored. Therefore, this study further examined the expression levels of nine genes highly expressed in fruit (*PmKAN8*, *PmKAN9*, *PmKAN14*, *PmKAN16*, *PmKAN17*, *PmKAN19*, *PmKAN20*, *PmKAN21*, and *PmKAN24*) across five key developmental stages of *P. mume*: fruit set phase (T1), primary expansion phase (T2), endocarp lignification phase (T3), secondary expansion phase (T4), and maturation phase (T5) (Figure 10).

The results revealed that the expression of *PmKAN8* and *PmKAN9* generally increased throughout development, peaking at the maturation stage. Concurrently, *PmKAN17* and *PmKAN20* also reached their highest expression levels at this same stage, albeit following different expression patterns. In contrast, *PmKAN14* was significantly upregulated during the pit hardening period. *PmKAN16* and *PmKAN24* exhibited bimodal expression patterns, with peaks occurring during the two rapid growth phases. *PmKAN19* and *PmKAN21* were highly expressed at T2 but declined thereafter. In summary, distinct expression patterns of *PmKAN* genes were observed across fruit developmental stages, suggesting their potential involvement in diverse regulatory mechanisms underlying different phases of fruit development.

## 3. Discussion

The *KANADI* transcription factor family, classified within the GARP subclass under the MYB-like, serves as a key regulator of lateral organ polarity establishment in plants. While systematic characterization of *KAN* homologs has been identified in several flowering plants—including *A. thaliana* with four members [2], *Nicotiana benthamiana* with eight [5], *Populus trichocarpa* with eight [4], and *Medicago truncatula* with five [20]—studies on their functions in woody perennial plants are still limited. Phylogenetic analysis of four Rosaceae species (*P. mume*, *P. armeniaca*, *P. salicina*, *F. vesca*) and the outgroup *A. thaliana* revealed significant expansion of the *KAN* gene family during Rosaceae evolution. Specifically, 26 *KAN* genes were identified in the *P. mume* genome, with substantial numbers also detected in other Rosaceae species (23 in *P. armeniaca*, 25 in *P. salicina*, and 19 in *F. vesca*). This expansion pattern suggests the occurrence of genomic duplication events in Rosaceae, a conclusion further supported by subsequent synteny analysis. Prior studies have demonstrated that whole-genome duplication events facilitated rapid diversification of Rosaceae species following major geological–climatic events [21]. Furthermore, studies have demonstrated that *AtKAN* genes participate in plant growth and development [4,22,23,24], exhibiting functional redundancy [1,12]. Phylogenetic analysis of *KAN* genes in this study revealed that *PmKAN4*, *PmKAN21*, and *PmKAN22* cluster with the four Arabidopsis *AtKAN* genes, suggesting potential functional similarity and redundancy among these three *P. mume* genes, which merits further investigation. The clustering of other *PmKAN* genes into distinct subgroups indicates divergence during *P. mume* evolution, as also suggested by the duplication events identified through chromosomal localization and synteny analysis.

Substantial physicochemical divergence was observed among *PmKAN* paralogs, with molecular weights ranging from 26.7 to 54.3 kDa and isoelectric points (pI) spanning 5.09~9.16, with differences may arise from real biological differentiation (e.g., functional evolution, structural variation) and these differentiation results further support the idea that *KAN* genes undergo species-specific adaptation, possibly driven by different environmental conditions and selection pressures. Temporal expression profiling across fruit developmental stages revealed dynamic regulatory patterns, providing critical insights into their roles in organogenesis. These findings establish a foundation for functional dissection of *KAN*-mediated regulatory networks in woody fruit species.

Secondary and tertiary structural analyses revealed conserved architectural features among *KAN* subfamily members, predominantly comprising coil regions and α-helix domains. A similar phenomenon was found in the structure prediction of the NAC proteins of *P. mume*, where each subfamily could elect a protein representative of the structural features of that subfamily, which possessed similar structural domains [25]. Conserved motif profiling identified analogous patterns within phylogenetic clades, consistent with putative functional conservation as documented in prior studies [6]. Differences in exon and intron structure within the *PmKAN* gene family showed clear patterns, with similar structures within each subfamily and variation between individual genes. This suggests that different *PmKAN* genes may have developed specialised functions. The chromosomal localisation analysis revealed that 26 *PmKAN* members are distributed across eight chromosomes, with three genes (*PmKAN8*, *PmKAN9* and *PmKAN10*) demonstrating tandem duplication events. The presence of intragenomic syntenic *PmKAN* gene pairs, coupled with interspecies genomic synteny analysis across *A. thaliana*, *P. mume*, *F. vesca*, *P. salicina*, and *P. armeniaca*, provides evidence for extensive gene duplication events in Rosaceae species. This mechanism fundamentally explains the substantial divergence in *KAN* gene numbers between these four Rosaceae species and *A. thaliana*. The Rosaceae-specific expansion of the *KAN* family implies its potential functional diversification in lineage-specific biological processes, such as complex floral organogenesis, fruit development regulation, and secondary growth formation. Statistical analyses further support the contribution of whole-genome duplication (WGD) to Rosid diversification [21,26]. Morphologically, certain WGD events correlate with key trait innovations, exemplified by the evolution of ovarian structures in the apple tribe (Maleae, Rosaceae) [21]. Following their most recent WGD, Maleae species developed a new type of fleshy fruit consisting of a fusion of the calyx and ovary (e.g., apple and pear) [26]. These taxa exhibit unique morphological characteristics and represent critical taxonomic units, indicating WGD’s role in driving clade-specific phenotypic innovation. The *KAN* gene expansion revealed in this study offers genomic insights into Rosaceae’s adaptive evolution. Future investigations should address the functional significance of *PmKAN* genes retained after WGD events in shaping floral and fruit diversification in *P. mume*.

Bioinformatic analysis of cis-regulatory elements in *PmKAN* promoters revealed that all 26 family members contained a high density of cis-regulatory elements associated with growth and development, light responsiveness, hormonal regulation, and stress adaptation. Previous studies have established *KANADI* transcription factors as critical regulators of organ polarity in leaves and pistils [2,27], leaf morphogenesis [4,13], and shoot apical meristem development [28,29,30], functioning through intricate interactions with phytohormones including auxin (IAA), gibberellins (GAs), and abscisic acid (ABA) [24,31,32,33,34]. Recent mechanistic insights from rice demonstrate that the *OsKAN1*–*OsYAB5* protein complex directly regulates *OsGA2ox6* expression to modulate bioactive gibberellin levels, thereby controlling plant height [16]. Combining our results and these previous studies suggest that *PmKAN* genes may orchestrate developmental processes and morphogenetic events through hormonal signalling pathways and metabolic regulation. However, the precise molecular mechanisms underlying *KAN*-mediated developmental regulation, particularly in woody species, remain poorly understood and present a pivotal research frontier for plant developmental biology.

The expression patterns of *PmKAN* genes across various tissues, as revealed by qRT-PCR analysis, exhibited distinct variation, consistent with findings reported for *KAN* families in other plant species [1,2,6]. Notably, among the 26 *PmKAN* genes, a significant number displayed markedly elevated expression in root (13 genes) and fruit (9 genes), implying their potential biological roles in these organs and warranting further functional investigation. Additionally, genes with relatively low expression levels may also encode transcriptional repressors, negative regulators of signaling pathways, or other critical regulatory molecules, and should not be overlooked in subsequent studies.

Previous studies on the *KAN* gene family have primarily focused on vegetative tissues—such as roots, stems, and leaves [6,20]—while research into their roles in reproductive organ development remains relatively limited. In rice, *SLL1* alleles (ah2/sll1) exhibit grain size defects through promoter binding of size-related genes, with mutants showing impaired cell cycle progression and proliferation [14]. Si [15] identified an *OsKANADI1* allele functioning as an extragenic suppressor of the temperature-sensitive osrdr6-1 mutant, which exhibited compromised tasiRNA biogenesis and lemma polarity. The osrdr6-1 hf1 double mutant displayed reduced grain size compared to both *osrdr6-1* and wild-type *ZH11*. As a key abaxial determinant, *OsKANADI1* transcriptionally activates the tasiR-ARFs pathway to maintain *OsARF3a* expression levels, thereby regulating lemma morphogenesis. These studies also provided additional evidence for future comprehensive research on the regulation of plant growth and development by the *KAN* gene family.

As a key reproductive organ of *P. mume*, the fruit is highly valued for its distinctive acidity and nutritional richness. However, research on *P. mume* fruit remains limited, and the role of *KAN* genes in this fruit has not yet been explored. In this study, expression profiling across different tissues identified nine genes (*PmKAN8*, *PmKAN9*, *PmKAN14*, *PmKAN16*, *PmKAN17*, *PmKAN19*, *PmKAN20*, *PmKAN21*, and *PmKAN24*) with high expression levels in fruit. Their expression dynamics were further examined during five critical developmental stages: fruit set phase (T1), primary expansion phase (T2), endocarp lignification phase (T3), secondary expansion phase (T4), and maturation phase (T5). RT-qPCR results revealed distinct expression patterns of these *PmKAN* genes across the developmental phases, suggesting that different *PmKAN* genes may play specialized roles at specific stages of fruit development. In conclusion, the molecular mechanisms through which *KAN* family genes regulate fruit development remain to be elucidated, warranting further in-depth investigation.

## 4. Materials and Methods

### 4.1. Plant Materials

**Figure 11:**
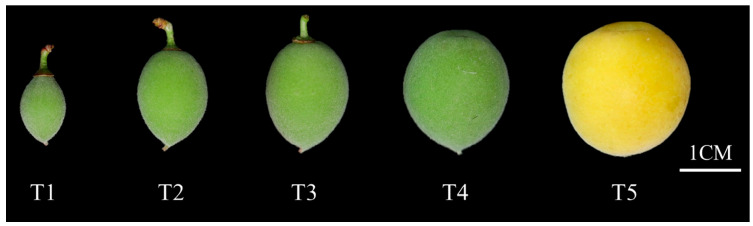
Fruit growth and development in ‘Xinnongxiaomei’. (T1) Fruit set phase, (T2) primary expansion phase, (T3) endocarp lignification phase, (T4) secondary expansion phase, (T5) maturation phase.

Plant materials were obtained from the National Field GeneBank for *P. mume* in Baima, Lishui, Nanjing, Jiangsu Province, China (Geographic coordinates 31°55′ N, 115°15′ E), using the cultivar ‘*Xinnongxiaomei*’. Roots, stems, leaves, buds, and mature fruits were simultaneously collected from one-year-old shoots of ten-year-old trees during February to May 2025. In a separate collection, fruit samples representing five distinct developmental stages were obtained (Figure 11): fruit set phase (T1), primary expansion phase (T2), endocarp lignification phase (T3), secondary expansion phase (T4), and maturation phase (T5). The experiment included three independent biological replicates. All specimens were immediately flash-frozen in liquid nitrogen and stored at −80 °C in ultra-low freezers for subsequent analyses.

### 4.2. Identification of PmKAN Family Members

Protein sequences and annotation files for *P. mume* were retrieved from NCBI (https://www.ncbi.nlm.nih.gov/, accessed: 20 October 2022). The PFAM (http://pfam.xfam.org/, accessed: 20 April 2024) database provided the *KAN* Hidden Markov Model (HMM) profile for screening candidate protein sequence data in the local *P. mume* proteome (E-value < 10^−5^) [19]. The *Fragaria vesca* genome reference is derived from octoploid cultivated strawberry [35], while the genomes of *Prunus* salicina and *Prunus armeniaca* were obtained from the online website (https://www.rosaceae.org/, accessed: 1 June 2025). In addition, the *Arabidopsis AtKAN1* sequence (TAIR, https://www.arabidopsis.org/, accessed: 21 April 2024) was used as a probe to search the *P. mume* protein database online using BlastP (https://ftp.ncbi.nlm.nih.gov/blast/executables/blast+/LATEST/, accessed: 26 April 2024). Protein sequences obtained by both methods were utilized for co-screening. TBtools (v2.0) facilitated sequence extraction and domain validation through InterPro (http://www.ebi.ac.uk/interpro/, accessed: 6 May 2024) and SMART with chromosomal mapping data concurrently acquired [36], with detailed steps refer to Zhang et al. [37]. Phylogenetic reconstruction was performed using the maximum likelihood (ML) method with IQ-TREE software (v2.3) [38]. The best-fit model for tree construction, JTT+F+R5, was selected using ModelFinder [39]. Bootstrap support values (1000 replicates) were calculated, and the resulting topology was visualized using iTOL (https://itol.embl.de/, accessed: 6 October 2024). The 26 identified *KAN* homologs, designated *PmKAN1*-*PmKAN26* based on chromosomal positions, underwent physicochemical characterization through ProtParam (https://web.expasy.org/protparam/, accessed: 22 October 2022), determining molecular weight, isoelectric point (pI), and GRAVY index. Referring to NetSurfP-3.0 [40] to predict the secondary protein structure of *PmKANs* with default parameters; the tertiary structure was constructed using the online website SWISS-MODEL (https://swissmodel.expasy.org/interactive, accessed: 20 June 2024) to build the structure model.

### 4.3. Analysis of Structure and Conserved Motifs of PmKAN Gene in P. mume

The gene structure diagrams of *PmKAN* family members were generated using the GSDS tool (https://gsds.gao-lab.org/) [41]. Conserved motifs were predicted by analysing *PmKAN* protein sequences with the MEME Suite (http://meme-suite.org/tools/meme, accessed: 22 October 2022), with motif lengths restricted to 6–50 residues and the number of motifs set to 10 based on structural domain integrity. Default parameters were retained for all other settings. Visualization of motif distributions was performed in TBtools using default configurations.

### 4.4. Chromosomal Location and Syntenic Analysis

The genomic localization of *PmKAN* genes was mapped using the *P. mume*.gtf annotation file and *PmKAN* gene list as input data in TBtools. Tandem and segmental duplication events within the *PmKAN* family were detected using MCScanX (https://github.com/wyp1125/MCScanX, accessed: 21 July 2024), implemented with default parameters for evolutionary divergence analysis. The MCScanX analysis involves constructing a gene family protein database with diamond, executing diamond blastp alignment (E-value = 1 × 10^−10^), and performing collinearity analysis of *KAN*s in *P. mume* using MCScanX, as well as collinearity analysis between *KAN* genes of *P. mume* and *Arabidopsis.*

### 4.5. Analysis of Cis-Acting Elements in PmKAN Gene Upstream Promoter Region

The 2000 bp genomic sequences upstream of *PmKAN* transcription start sites were extracted from *P. mume* for cis-acting element analysis, and plant material was obtained from young leaves of annual branches of ten-year-old fruit trees. Putative regulatory elements were predicted using PlantCARE (http://bioinformatics.psb.ugent.be/webtools/plantcare/html/, accessed: 17 May 2024) [42], followed by functional categorization. To resolve evolutionary relationships, protein sequences were aligned using the MAFFT (v7.0) [43], and a maximum likelihood (ML) phylogenetic tree was reconstructed with IQ-TREE. The optimal substitution model (VT+F+R4) was selected via ModelFinder (v2.0) and applied to ML inference. The IQ-TREE is employed to improve the Bootstrap parameter, set at 1000.

### 4.6. Quantitative qRT-PCR Analysis of PmKAN Genes

RNA was extracted using the Tiangen RNA extraction kit (DP441) (Beijing, China) from various tissues (root, stem, leaf, bud, and fruit) of the ‘*Xinnongxiaomei*’ cultivar of *P. mume*, with each sample weighing 50–100 mg. First-strand cDNA synthesis was carried out with the PrimeScript RT reagent Kit with gDNA Eraser (Perfect Real Time) (Takara, Beijing, China) following manufacturer specifications. Subsequent quantitative PCR analysis was conducted on the QuantStudio 5 Flex platform (Applied Biosystems, Waltham, MA, USA) employing SYBR Green chemistry. The 20 μL reaction system comprised 10 μL SYBR Green qPCR Supermix (AG11701; Accurate Biology, Hunan, Changsha, China), 1 μL each of forward and reverse primers (10 μM), 1 μL cDNA template (5 ng/μL), and 7 μL nuclease-free water. Thermal cycling parameters consisted of initial denaturation at 95 °C for 3 min, followed by 40 cycles of 95 °C denaturation (15 s) and 60 °C combined annealing/extension (30 s). Fluorescence acquisition occurred during the annealing/extension phase, with subsequent melting curve verification. Relative gene expression was calculated using 2^−ΔΔCt^ method, with values normalised to the actin reference gene. The primer sequences used are listed in Table 3. Three biological samples, each with three technical repeats, were used to ensure reliable results [44].

## 5. Conclusions

This study identified 26 *KAN* family genes in *P. mume* using bioinformatic approaches. Phylogenetic classification clustered these *PmKAN* genes into five subfamilies, with members within each subfamily sharing conserved motif patterns and protein structures, suggesting functional conservation. Evolutionary analysis incorporating *P. mume*, related species (*P. armeniaca*, *P. salicina*, *F. vesca*), and the outgroup *A. thaliana* revealed whole-genome duplication events in Rosaceae that drove substantial *KAN* gene expansion, potentially facilitating trait diversification and adaptive evolution. Promoter analysis and expression profiling indicated *PmKAN* genes are involved in developmental and metabolic regulation, highlighting the need for further functional characterization. The qRT-PCR analysis revealed predominant expression of *PmKAN* genes in root and fruit tissues. Further investigation of the fruit enriched genes suggests their potential involvement in stage specific regulatory functions during fruit development. In conclusion, genome-wide identification of the *KAN* family facilitates understanding of the evolution and function of this gene family in *P. mume*, establishes a molecular framework for studying its role in Rosaceae plants, and provides important expression evidence and clues for deciphering the functional differentiation of *PmKAN* genes in *P. mume*.

## Figures and Tables

**Table 1:** Characterization of *KAN* gene family in *P. mume*.

Sequence ID	Gene ID	Protein ID	Number of Amino Acid	Molecular Weight (Da)	Theoretical pI	Instability Index	Aliphatic Index	Grand Average of Hydropathicity
*PmKAN*1	LOC103341420	XP_008243158.1	339	38,330.28	7.22	54.77	66.17	−0.996
*PmKAN*2	LOC103342254	XP_008244092.2	256	28,978.03	9.16	54.78	78.55	−0.628
*PmKAN*3	LOC103318708	XP_008218346.1	458	51,491.82	5.09	60.69	62.14	−0.941
*PmKAN*4	LOC103320783	XP_008220727.1	323	35,643.63	8.38	40.90	63.72	−0.729
*PmKAN*5	LOC103321009	XP_008220974.1	300	33,444.13	8.86	62.06	62.43	−0.782
*PmKAN*6	LOC103321568	XP_008221605.1	346	38,907.83	7.68	46.67	74.62	−0.810
*PmKAN*7	LOC103324237	XP_008224497.1	311	33,616.20	6.11	52.42	81.03	−0.352
*PmKAN*8	LOC103324390	XP_008224659.1	305	33,185.51	6.50	44.14	71.97	−0.551
*PmKAN*9	LOC103324391	XP_008224662.1	306	33,843.00	6.22	58.46	73.66	−0.744
*PmKAN*10	LOC103324465	XP_008224747.1	240	26,712.18	8.25	51.90	75.96	−0.642
*PmKAN*11	LOC103324956	XP_008225297.1	417	48,261.83	8.84	60.77	58.39	−1.06
*PmKAN*12	LOC103325588	XP_008226001.1	368	40,994.31	8.55	55.89	65.49	−0.787
*PmKAN*13	LOC103327465	XP_008228027.1	466	51,018.80	5.58	75.71	69.10	−0.688
*PmKAN*14	LOC103328312	XP_008228924.1	432	49,087.78	8.70	52.45	68.56	−0.811
*PmKAN*15	LOC103328941	XP_016649340.1	240	27,684.77	6.60	48.97	60.92	−0.890
*PmKAN*16	LOC103329105	XP_008229756.1	422	47,845.56	5.09	52.08	77.16	−0.646
*PmKAN*17	LOC103332813	XP_008233790.1	360	40,640.07	6.77	49.14	61.17	−0.946
*PmKAN*18	LOC103334420	XP_016650539.1	496	54,847.79	5.44	61.42	70.58	−0.626
*PmKAN*19	LOC103335407	XP_008236633.1	394	44,002.27	7.61	58.71	62.64	−0.831
*PmKAN*20	LOC103336798	XP_008238120.1	278	31,421.01	8.30	37.42	62.12	−0.981
*PmKAN*21	LOC103336839	XP_008238177.1	409	45,952.91	7.39	70.15	65.82	−0.796
*PmKAN*22	LOC103338908	XP_008240401.1	470	52,192.55	6.78	54.37	57.94	−0.829
*PmKAN*23	LOC103338947	XP_008240441.1	438	49,015.82	7.36	49.74	69.06	−0.817
*PmKAN*24	LOC103339100	XP_008240600.1	289	31,469.29	7.69	53.84	65.78	−0.792
*PmKAN*25	LOC103339429	XP_008240946.2	419	47,156.13	7.66	70.79	64.25	−0.819
*PmKAN*26	LOC103341001	XP_008242700.1	496	54,334.02	7.15	62.68	56.94	−0.882

**Table 2:** Prediction of secondary structure of KAN protein in *P. mume*.

Protein Name	Coil Regions	310 Helix (%)	Alpha Helix (%)	Random Coil (%)	Beta Strand (%)	Beta Turn (%)
PmKAN1	86.14	0.88	10.62	0.88	0.88	0.59
PmKAN2	70.70	0.78	26.56	1.95	0.00	0.00
PmKAN3	82.53	0.66	15.94	0.87	0.00	0.00
PmKAN4	87.00	0.93	10.22	0.62	1.24	0.00
PmKAN5	86.00	1.00	11.00	1.00	0.33	0.67
PmKAN6	72.83	0.58	25.43	1.16	0.00	0.00
PmKAN7	68.49	0.64	28.62	1.93	0.32	0.00
PmKAN8	69.84	0.66	26.89	1.97	0.66	0.00
PmKAN9	71.24	0.98	26.14	1.63	0.00	0.00
PmKAN10	80.42	1.25	13.33	1.67	2.50	0.83
PmKAN11	88.97	0.72	8.39	0.72	0.72	0.48
PmKAN12	78.26	0.54	20.11	1.09	0.00	0.00
PmKAN13	80.90	0.00	17.38	1.72	0.00	0.00
PmKAN14	90.05	0.69	7.41	0.69	0.69	0.46
PmKAN15	82.08	0.83	13.33	2.08	0.83	0.83
PmKAN16	72.75	0.71	25.12	0.95	0.47	0.00
PmKAN17	86.39	0.83	10.00	0.83	1.39	0.56
PmKAN18	81.25	0.40	17.34	1.01	0.00	0.00
PmKAN19	73.10	1.02	23.35	1.27	1.27	0.00
PmKAN20	68.71	0.72	27.70	2.16	0.72	0.00
PmKAN21	89.98	0.73	7.82	0.00	0.98	0.49
PmKAN22	90.85	0.64	6.81	0.43	0.85	0.43
PmKAN23	77.63	0.46	20.55	1.14	0.23	0.00
PmKAN24	69.90	0.69	27.34	2.08	0.00	0.00
PmKAN25	79.24	0.48	18.85	1.43	0.00	0.00
PmKAN26	79.84	0.60	17.14	0.81	1.61	0.00

**Table 3:** Primer sequences used in qRT-PCR.

Gene Name	Forward Primer (5′→3′)	Reverse Primer (5′→3′)
*PmKAN 1*	TTCTCGCGGATCATCAAGGC	GAGGTTTGTTACCTGAAGCTGG
*PmKAN 2*	ACTTACGACGTTGCACCGAA	TCGTTTTCCACAGAAGGCAA
*PmKAN 3*	GCAGAAGGCACAAGAAACCG	GGCTGGGTACCAGACTCAAC
*PmKAN 4*	CCGCAATCAAATGGCACCAA	ACAAGGACAATAATGCGCACAC
*PmKAN 5*	CCTTCTGAGTATGACCAGCGA	AGGCAATAGAGGTGACGCAG
*PmKAN 6*	TGGAAGTGCAGAGGAAACTTCA	AGAACTCTGGGTTTGTTGGCA
*PmKAN 7*	GCATGAGCAGCTGGAGGTTA	GGTGGTTTTTGGTCATGCGG
*PmKAN 8*	GTCAGCTTGGCGGTTCATCT	CATCGCCCATTTCCTTCCCT
*PmKAN 9*	GCGACTGCACGAGCAATTAG	AGAGCCAGGTGCTTCTGAAC
*PmKAN 10*	GCTGCTATGTCTCTCTGGGC	CTGCACTCACTAGCGACACA
*PmKAN 11*	ACTTGCCTCAATCCTTCTTCCA	CTCTCCCCTTTCACCGCATC
*PmKAN 12*	GAGGTTGCTTTCCGAGCCTA	TTTTCGGTTTGCCTTGACGA
*PmKAN 13*	TCATCAGCTGGTGTATCGCC	GGTGGCTCAGATTCACTGCT
*PmKAN 14*	GTCTTGTCAAGTCGTGTAAGC	CGCAAAAGCAAGGACTAAAATGC
*PmKAN 15*	CAAAAGAGGAAGCAGCAGGC	TCAGTTGCAGACCACGACAG
*PmKAN 16*	CCTCCTAGGCCTGGACAGTT	CACGAATAACTCCCCCAGCA
*PmKAN 17*	CTTAGGTGTTGCTCCTGCAC	TTAATTTGCCTCTGCCCAGC
*PmKAN 18*	GGGGGTATCTGCAGCAATGT	GACACCTGTTGGGAGTCAGG
*PmKAN 19*	AATAGGGCTGCTGGAAGAGC	TTATCTTCCTTCCTCGCCGC
*PmKAN 20*	CGGACTTGGGAGTTGCTTCT	GGTGATCAATTTGTCGTTTTGGC
*PmKAN 21*	AGGAGTGAGAGAGTAGAGCTTG	GCGCTGGACTACCACTCTTC
*PmKAN 22*	GGAGGGGCTGACTTTCATGG	ACAAGAGGAACATGAGGGCG
*PmKAN 23*	GGAGTAGCCTCTGCATTGGA	AACCAGCTAGGAGGAGCAGA
*PmKAN 24*	GAAAAGCCCAACGCCTTCTG	GACGAAGCGGTCATGGAGAT
*PmKAN 25*	AGCACTTTCATATTGCCGAGG	TGAGCTGCTTCCCTTGTTCTT
*PmKAN 26*	GGTTGACGGTTTGACCAACG	ATGTTTCATGCTGCCAGTGC
*PmACTIN*	TGAAGCATACACCTATGATGATGAAG	CTTTGACAGCACCAGTAGATTCC

## Data Availability

Data is contained within the article.

## Abbreviations

The following abbreviations are used in this manuscript:

ABA	Abscisic acid
CTK	Cytokinin
FPKM	Fragments Per Kilobase of transcript per Million mapped fragments
GA	Gibberellic acid
HMM	Hidden Markov Model
JA	Jasmonic acid
KAN	KANADI
MeJA	Methyl jasmonate
MEME	Multiple Em for Motif Elicitation
ML	Maximum Likelihood
NCBI	National Center for Biotechnology Information
ORF	Open Reading Frame
*P. mume*	*Prunus mume* Sieb. et Zucc.
PFAM	Protein Family Database
SA	Salicylic acid
WGD	Whole-Genome Duplication
